# PEG-Infiltrated Polyoxometalate Frameworks with Flexible Form-Factors

**DOI:** 10.1021/acsmaterialslett.2c00393

**Published:** 2022-09-06

**Authors:** Liana S. Alves, Linfeng Chen, Carl E. Lemmon, Milan Gembicky, Mingjie Xu, Alina M. Schimpf

**Affiliations:** † Department of Chemistry and Biochemistry, University of California, San Diego, La Jolla, California 92093, United States; ‡ Irvine Materials Research Institute, University of California, Irvine, California 92697, United States

## Abstract

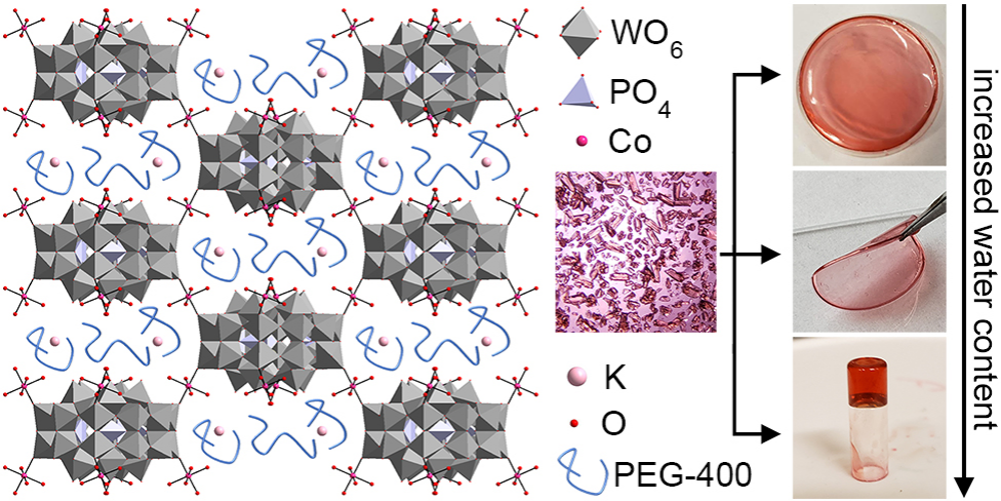

We present the synthesis of metal oxide frameworks composed of the Preyssler anion, [NaP_5_W_30_O_110_]^14–^, bridged with transition-metal cations and infiltrated with polyethylene glycol. The frameworks can be dissolved in water to form freestanding rigid or flexible films or gels. Powder X-ray diffraction shows that all form-factors maintain the short-range order of the original crystals. Raman spectroscopy reveals that, similar to hydrogels, the macroscopic mechanical properties of these composites are dependent on the water content and the extent of hydrogen-bonding within the water network. The understanding gained from these studies facilitates solution-phase processing of polyoxometalate frameworks into flexible form factors.

The construction of extended networks from molecular clusters has recently gained attention as a strategy for synthesizing new materials with precisely tailored atomic positions and rationally designed properties.^1−12^ Polyoxometalates (POMs) are well-suited as anionic ligands for coordination networks because of their oxygen-rich surface, providing multiple coordination sites. Furthermore, POMs have immense structural and compositional variability, giving rise to unique electronic, magnetic, or photophysical properties,^13−17^ as well as to rich, reversible redox activity.^13,14,17−19^ Indeed, the assembly of POMs into coordination networks or other suprastructures has been increasingly used to access complex metal-oxide materials with diverse structures and functionalities.^3,5−7,20−38^

The synthesis of POMs and POM-based networks is usually performed to yield high-quality crystals or polycrystalline powders, but such form factors are not inherently suitable for many applications and may not be easily solution-processed. Advances in metal–organic framework research have enabled them to be processed into flexible form-factors through combination with polymers, often in mixed-matrix membranes.^39−41^ Recently, POM–polymer composites have been used to combine the exciting properties of POMs with the facile processability and ductile nature of organic polymers, yielding hybrid materials with new functionalities.^42−46^ A common strategy for composite formation is post-synthetic physical blending, but such mixtures are not held together well and can phase-segregate. Alternatively, electrostatic interactions have been used to promote composite assemblies, but these methods may be difficult to scale and are limited to charged polymers. Furthermore, these strategies have not been utilized for ordered, extended POM networks. Covalent functionalization of POMs has been used to access hybrid materials and networks, but these methods can be synthetically challenging and are not viable for all POMs.

We report the synthesis of a polymer-infiltrated POM framework, crystals of which can be processed into various form-factors. Specifically, a framework composed of the Preyssler anion, [NaP_5_W_30_O_110_]^14–^ (denoted as {P_5_W_30_}),^47^ bridged with transition-metal ions and infiltrated with polyethylene glycol (PEG) is presented (Figure 1). Crystals of these frameworks show remarkable stability toward desolvation, compared to those without PEG. Importantly, the crystals can be dissolved in water to form gels or to be recast as films, with all form-factors displaying short-range order analogous to that of the original crystals. Electron microscopy images reveal that, unlike physically blended composites, films presented herein are homogeneous on the submicrometer scale. The mechanical properties of the films are dependent on the humidity, allowing for reversible switching between rigid and flexible states. Using Raman spectroscopy, we show that increased flexibility is due to higher water content, which corresponds to a decrease in hydrogen bonding within these framework–PEG–water composites. These experiments elucidate the factors important to achieving flexible form-factors with POM-based frameworks and ultimately facilitate their solution-phase processing for a wide range of applications.

**Figure 1:**
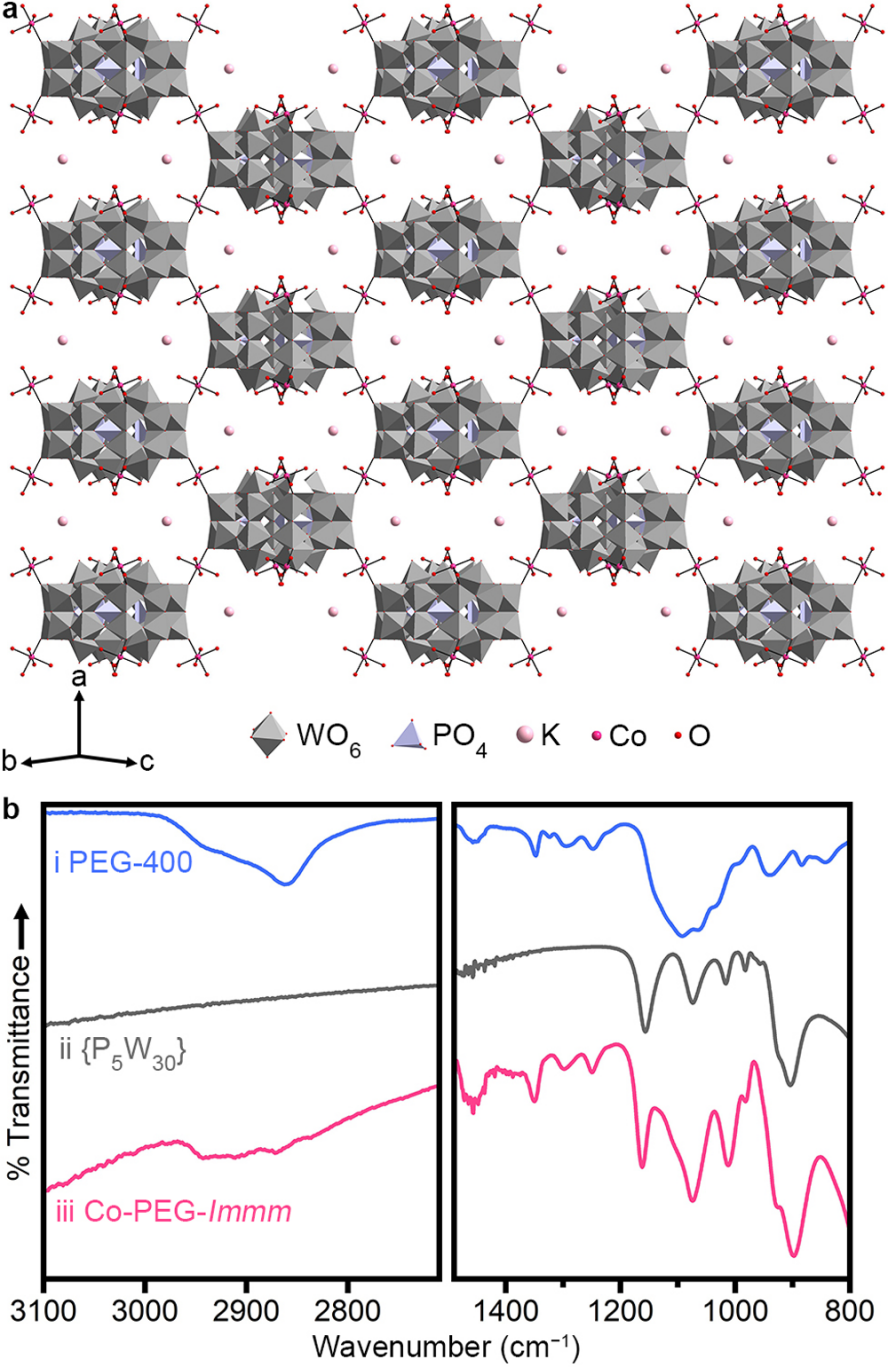
(a) Crystal structure of PEG-containing Co-bridged {P_5_W_30_} (Co-PEG-*Immm*). (b) IR absorption spectra of (i) PEG-400, (ii) {P_5_W_30_}, and (iii) crushed, washed crystals of Co-PEG-*Immm*.

PEG-containing frameworks (Figure 1) were synthesized using the same methods as used for non-PEG frameworks,^36−38^ with the addition of PEG during the crystallization stage. Briefly, K_14–*x*_Na_*x*_[NaP_5_W_30_O_110_] and CoCl_2_·6H_2_O were added to 1 M aqueous LiCl and the solution was refluxed for ∼12 h. Upon cooling to room temperature, PEG-400 (120 equiv/{P_5_W_30_}) was added to the solution and crystals were grown via methanol (MeOH) diffusion into the solution. The resulting crystals have a structure that is distinct from those obtained without PEG.^36−38^Figure 1a shows the crystal structure of frameworks synthesized with CoCl_2_·6H_2_O, which yielded pink crystals with an orthorhombic *Immm* unit cell (Co-PEG-*Immm*; Table S1; *a* = 17.9869(7) Å, *b* = 21.8213(8) Å, and *c* = 24.8909(10) Å). Each {P_5_W_30_} is connected to eight, crystallographically equivalent neighboring clusters through the Co(H_2_O)_4_^2+^ bridging ions (Figures 1a and S1). Electron density in the void space of the structure is assigned to K^+^ (2 per {P_5_W_30_}), which is likely coordinated by a combination O from PEG and water.

Although PEG cannot be assigned crystallographically, IR spectroscopy reveals that the polymer is present even after the crystals are crushed and extensively washed (Figure 1b), suggesting that PEG incorporates into the void space of the framework. It is likely that the PEG wraps around the K^+^ within the pores.^48^ Importantly, inclusion of PEG imparts additional stability of the framework against desolvation. Unlike our previous {P_5_W_30_}-based frameworks, which contract upon removal from the mother liquor,^36^ the structure of Co-PEG-*Immm* is largely unchanged upon removal from the mother liquor (Figure S2). Elemental analysis was used to estimate a formula of H_1.5_Li_1.5_NaK_2_Co_4_[NaP_5_W_30_O_110_]·3PEG·24.5H_2_O. This polymer content is higher than most {P_5_W_30_}-based composites,^49−52^ but is consistent with the void space of the framework. Based on the crystal structure, the void space is calculated to be ∼2500 Å^3^ per {P_5_W_30_}, which could fit up to ∼4 PEG-400 molecules (Table S2). Crystallization with larger polymers that do not fit in the void space of the framework did not readily yield polymer-infiltrated crystals.

We note that the structure presented in Figure 1a is simplified by showing only one of the two disordered cluster configurations. In addition, cations found in elemental analysis cannot be assigned crystallographically because of large disorder. This high level of disorder is typical of POM-based coordination networks and is seen in some previous {P_5_W_30_} frameworks.^36−38^

Analogous synthetic conditions can be used to obtain isostructural *Immm* frameworks with M(H_2_O)_4_^*n*+^ (M^*n*+^ = Mn^2+^, Fe^2+/3+^, Ni^2+^, Zn^2+^) bridging ions (Table S1, Figures S3 and S4). When the same synthesis was performed with CuCl_2_·6H_2_O, however, isostructural frameworks were not initially obtained (Figure S3). Instead, the resulting crystals are composed of Cu(H_2_O)_5_^2+^-decorated {P_5_W_30_} with an orthorhombic *C*222_1_ unit cell (Table S3 and Figure S5). IR spectroscopy of crushed and washed crystals revealed that all (M(H_2_O)_4_^*n*+^-bridged and Cu(H_2_O)_5_^2+^-decorated) contained PEG (Figure S5).

Importantly, the incorporation of PEG into these {P_5_W_30_}-based frameworks enables facile processing of the framework architecture into various form-factors. When crystals of Co-PEG-*Immm* (Figure 2a, trace i) were dissolved in water, the resulting solution could be drop-cast into films that are rigid (Figure 2a, trace ii) or flexible (Figure 2a, trace iii), depending on the humidity (<60% for rigid films, 60%–85% for flexible films). The films are free-standing and can be reversibly switched between the rigid and flexible forms using a humidity chamber or heat. Both the rigid and flexible forms maintain the short-range order found in the original crystals (Figure 2a). This ordering can also be seen in medium-angle annular dark field scanning transmission electron microscopy (MAADF-STEM) images of a rigid film (Figure S6). We note that PEG-400 is a liquid, and thus the films cannot simply be microcrystallites embedded in the PEG matrix. This claim is corroborated by scanning electron microscopy (SEM) imaging, which reveals that the films are homogeneous on the submicrometer scale (Figure S7).

**Figure 2:**
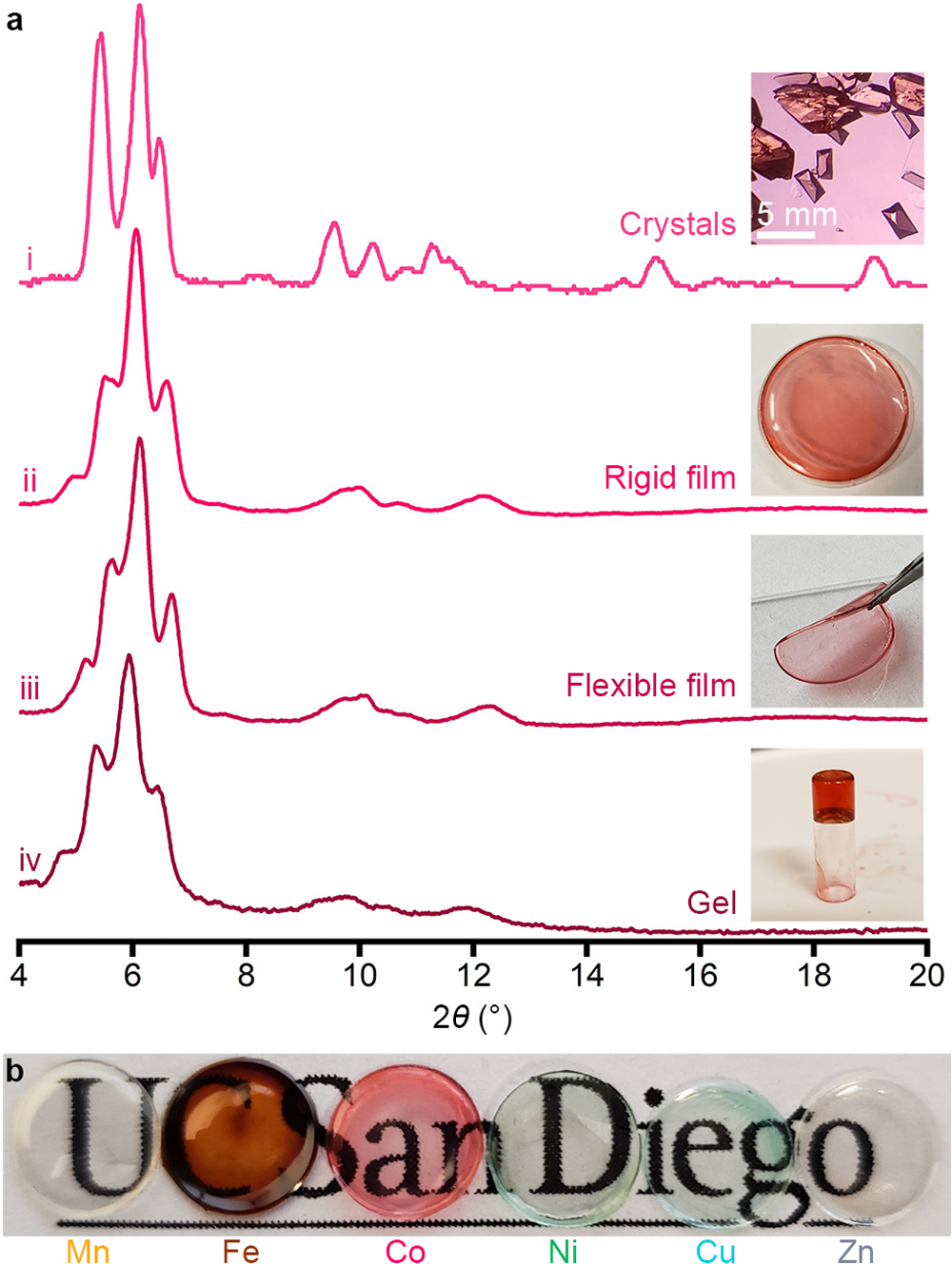
(a) Powder X-ray diffraction patterns and photographs of (i) Co-PEG-*Immm* crystals, Co-PEG-*Immm* cast into a film at (ii) ∼50% humidity (rigid, film diameter of ∼35 mm) and (iii) ∼60% humidity (flexible, film diameter of ∼15 mm), and (iv) Co-PEG-*Immm* dissolved in ∼50 equiv water and heated to form a gel. Insets show photographs of each form factor. (b) Photograph of PEG-containing (left to right) Mn-, Fe-, Co-, Ni-, Cu-, and Zn-bridged {P_5_W_30_} cast into films at ∼50% humidity. Each film is ∼8 mm in diameter.

Films could also be cast from other M-PEG-*Immm* frameworks (Figure 2b; M = Mn, Fe, Ni, Zn), which all show the same diffraction as that of films cast from Co-PEG-*Immm* (Figure S8). Interestingly, films cast from Cu-decorated clusters (Figure 2b) also show the same ordering as those cast from M-PEG-*Immm* frameworks (Figure S8).

The dependence of macroscopic mechanical properties on the humidity (i.e., water-content) is reminiscent of hydrogels, although our materials would dissolve if submerged in water. Indeed, the dissolution of concentrated Co-PEG-*Immm* forms a gel-like substance that does not flow but has short-range order similar to that of the crystals and films (Figure 2a, trace iv). The gel-like behavior of this form-factor was verified using parallel-plate rheological measurements. Figure 3 shows the storage (*G*′, closed circles) and loss (*G*″, open circles) moduli as a function of angular frequency (ω) measured at 2.6% strain. The observation of relatively flat moduli with *G*′ > *G*′′ confirms the gel-like nature under these measurement conditions, although with a relatively low ratio of *G*′/*G*′′.^53−59^ These gels are unique from previously reported “gel-like” POM–polymer coacervates, which did not diffract and behaved as viscoelastic liquids.^60^ Solid- and rubber-like composites have been formed with polyoxovanadates and gelatin, but do not contain an ordered, extended structure.^61^

**Figure 3:**
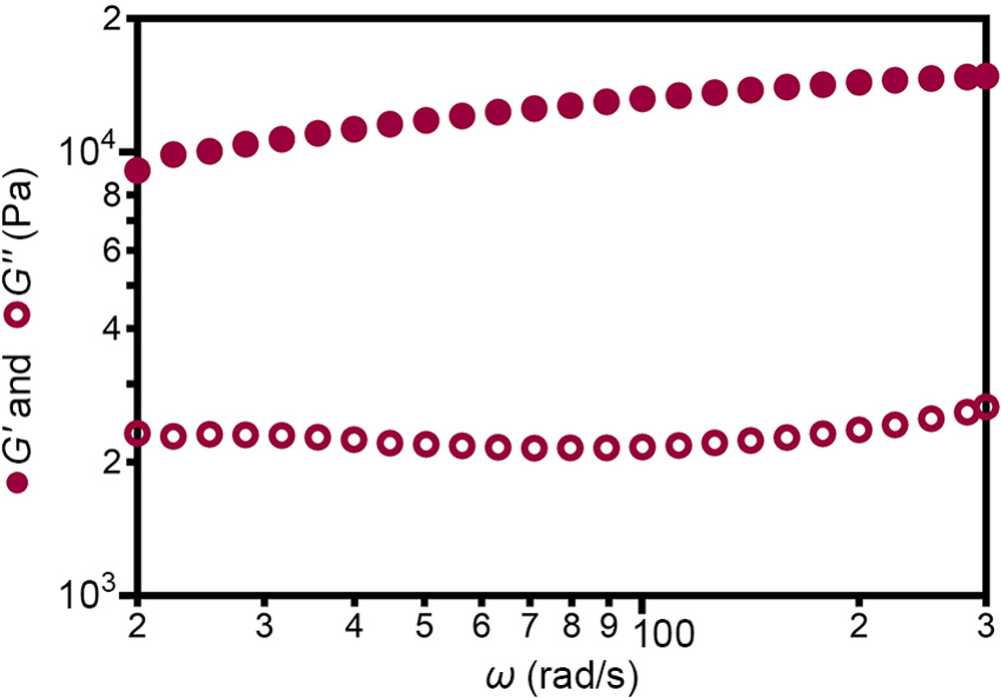
Storage (*G*′, closed circles) and loss (*G*″, open circles) moduli of the gel form-factor as a function of angular frequency (ω).

To evaluate the importance of the various components in accessing the varied form-factors, several controls were performed. First, PEG-free frameworks (Co-*Imma*)^36,37^ could not be cast into films using the same method, but instead resulted in a polycrystalline powder (Figure S9a). Similarly, we were unable to form homogeneous films from Co-*Imma* frameworks dissolved in water and mixed with PEG (Figure S9b), or from an aqueous mixture of CoCl_2_, {P_5_W_30_}, and PEG (Figure S9c). Finally, we synthesized PEG-infiltrated {P_5_W_30_} crystals (PEG-{P_5_W_30_}, Table S3 and Figure S10). When these crystals were dissolved in water, they could be cast into free-standing rigid films (Figure S10) with ordering different than the parent crystals. However, these films are not transparent and cannot be made flexible. Instead, increased humidity causes the films to break apart and eventually dissolve. These controls highlight the importance of the Co-bridged framework structure as well as the PEG in enabling access to various form-factors that maintain structural integrity. Indeed, the coordination mode of countercations was recently shown to play a crucial role in the formation of POM-based organogels.^62^

An important factor in the formation and mechanical properties of polymer-based hydrogels is the hydrogen-bonding network of the water molecules, which can be monitored using Raman spectroscopy.^63−67^Figure 4 shows the Raman spectra of the various form-factors of Co-PEG-*Immm*. In these spectra, the peaks at ∼2700–3000 cm^–1^ are assigned to the C–H modes of PEG^68^ and the intensity at ∼3100–3700 cm^–1^ is due to several overlapping water modes (Table S4, vide infra).^65−67,69−72^ Since each form-factor contains the same amount of PEG, the spectra are normalized to the C–H modes of PEG. Based on the envelope of water modes, the relative water content increases as crystals ≈ rigid film < flexible film < gel.

**Figure 4:**
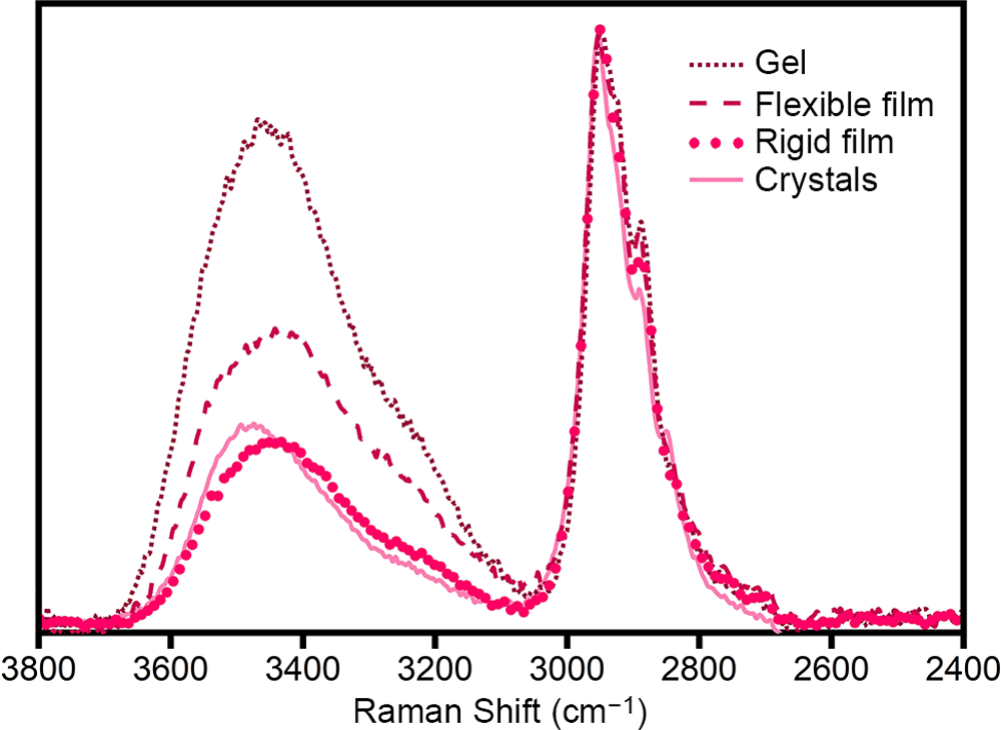
Raman spectra of Co-PEG-*Immm* crystals and of the flexible film, rigid film, and gel forms. Spectra are normalized to the C–H modes of PEG (∼2900 cm^–1^).

The envelope of water modes in the Raman spectra can be further analyzed to determine the relative amount of hydrogen bonding. The Raman spectrum of pure H_2_O is shown in Figure 5, trace i. This spectrum contains several water modes for strongly hydrogen-bound and weakly/non-hydrogen-bound water (Table S4).^65−67,69−72^ The dashed line at 3460 cm^–1^ is the isosbestic point, at which the Raman scattering is insensitive to the change in the amount/strength of hydrogen bonding.^69^ In other words, this line demarcates the strongly and weakly/non-hydrogen-bonding regimes. An increase in intensity to the right of this line (lower wavenumber) is indicative of greater hydrogen bonding, while increased intensity to the left (higher wavenumber) is indicative of lesser hydrogen bonding. To compare the amount/strength of hydrogen bonding between samples, the ratios of integrated intensities of the strongly hydrogen-bound region (3100–3460 cm^–1^) and weakly/non-hydrogen-bound (3460–3750 cm^–1^) were used (Table S5). From these data, it can be seen that the gel (Figure 5, trace ii), flexible film (Figure 5, trace iii), and rigid film (Figure 5, trace iv) have increased hydrogen bonding, compared to the parent crystals (Figure 5, trace v). Furthermore, the hydrogen bonding increases as gel < flexible film < rigid film. This trend is consistent with that observed in the swelling of polymer-based hydrogels, where increased water is added as “free” water, leading to an overall decrease in the ratio of hydrogen-bound/non-hydrogen-bound water.^65−67^ Thus, the hydrogel-like forms of Co-PEG-*Immm* show an increase in hydrogen bonding, relative to their parent crystals. In contrast, this increase in hydrogen bonding is not seen in the rigid PEG-{P_5_W_30_} film (Figure 5, trace vi) compared to the parent PEG-{P_5_W_30_} crystals (Figure 5, trace vii). Although the Co-PEG-*Immm* and PEG-{P_5_W_30_} crystals have similar levels of hydrogen bonding, film formation from PEG-{P_5_W_30_} does not lead to an increase in hydrogen bonding, preventing access to flexible form-factors. Similarly, no increase in hydrogen bonding is seen when we attempt to cast films (Co-*Imma* control, Figure 5, trace viii; Figure S9) from Co-*Imma* crystals (Figure 5, trace ix), which do not contain PEG. We note that both Co-PEG-*Immm* and PEG-{P_5_W_30_} crystals contain more hydrogen bonding than Co-*Imma* crystals, highlighting the importance of PEG in enabling the formation of the hydrogen-bound water-network. Overall, these data suggest that the many components of the Co^2+^-bridged frameworks are important for accessing the increased hydrogen bonding that enables flexible and switchable form-factors.

**Figure 5:**
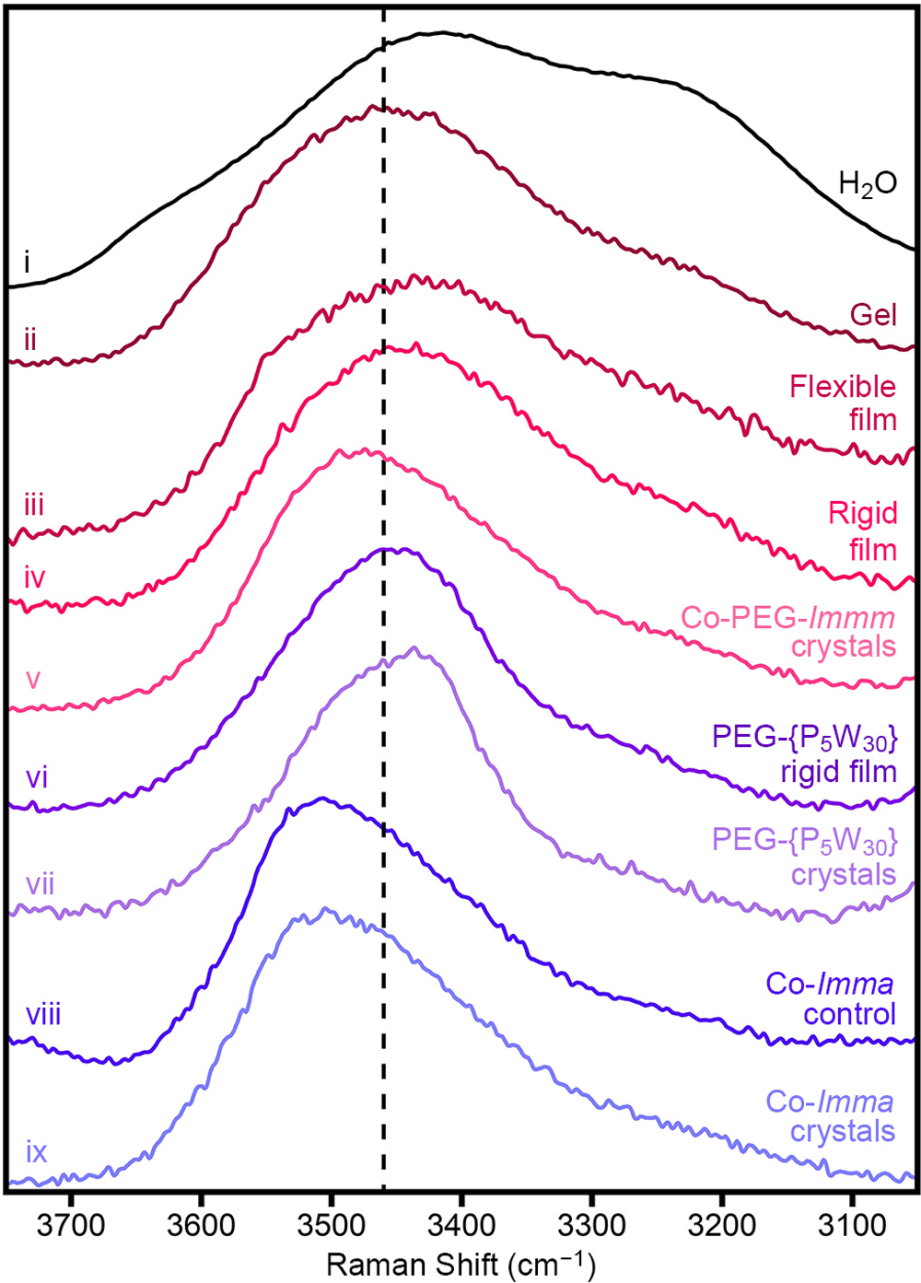
Raman spectra of (i) water, (ii) gel, (iii) flexible film, and (iv) rigid film forms derived from (v) Co-PEG-*Immm* crystals. (vi) rigid film cast from (vii) PEG-{P_5_W_30_} crystals. Control attempts to cast a (viii) film from (ix) Co-*Imma* crystals. The dashed vertical line indicates the isosbestic point for strongly versus weakly/non-hydrogen-bound water.

In summary, we have demonstrated that transition-metal bridged {P_5_W_30_} frameworks can be infiltrated with PEG to (i) imbue increased stability toward desolvation and (ii) enable facile, solution-phase processing into form-factors with various macroscopic mechanical properties. Similar to hydrogels, the flexibility of these materials is dependent on the amount of water trapped in the composites and on the extent of hydrogen bonding within the water network. These experiments elucidate factors that enable solution-phase processing of polyoxometalate-based frameworks into various form-factors.

## Supporting Information Available

Supplementary tables and figures; experimental details including synthesis and characterization (PDF)
Structural data for the PEG-infiltrated, Co-bridged framework H_*z*_Li_*y*_Na_*x*_K_6–*x*–*y*–*z*_Co_4_[NaP_5_W_30_O_110_]**·***m*PEG**·***n*H_2_O (Co-PEG-*Immm*) (CIF)
Structural data for the PEG-infiltrated, Mn-bridged framework H_*z*_Li_*y*_Na_*x*_K_5–*x*–*y*–*z*_Mn_4.5_[NaP_5_W_30_O_110_]**·***m*PEG**·***n*H_2_O (Mn-PEG-*Immm*) (CIF)
Structural data for the PEG-infiltrated, Fe-bridged framework H_*z*_Li_*y*_Na_*x*_K_5–*x*–*y*–*z*_Fe_4.5_[NaP_5_W_30_O_110_]**·***m*PEG**·***n*H_2_O (Fe-PEG-*Immm*) (CIF)
Structural data for the PEG-infiltrated, Ni-bridged framework H_*z*_Li_*y*_Na_*x*_K_5–*x*–*y*–*z*_Ni_4.5_[NaP_5_W_30_O_110_]**·***m*PEG**·***n*H_2_O (Ni-PEG-*Immm*) (CIF)
Structural data for the PEG-infiltrated, Zn-bridged framework H_*z*_Li_*y*_Na_*x*_K_6–*x*–*y*–*z*_Zn_4_[NaP_5_W_30_O_110_]**·***m*PEG**·***n*H_2_O (Zn-PEG-*Immm*) (CIF)
Structural data for the PEG-infiltrated, Cu-decorated Preyssler H_*z*_Li_*y*_Na_*x*_K_6–*x*–*y*–*z*_Cu_4_[NaP_5_W_30_O_110_]**·***m*PEG**·***n*H_2_O (Cu-PEG-*C*222_1_) (CIF)
Structural data for the PEG-infiltrated Preyssler H_*z*_Li_*y*_Na_*x*_K_14–*x*–*y*–*z*_[NaP_5_W_30_O_110_]**·***m*PEG**·***n*H_2_O (PEG-{P_5_W_30_}) (CIF)

## Supplementary Material

tz2c00393_si_001.pdf

tz2c00393_si_002.cif

tz2c00393_si_003.cif

tz2c00393_si_004.cif

tz2c00393_si_005.cif

tz2c00393_si_006.cif

tz2c00393_si_007.cif

tz2c00393_si_008.cif

## Accession Codes

Cambridge Structural Database codes for all deposited CIFs are reported in Tables S1 and S3. These data can be obtained free of charge via www.ccdc.cam.ac.uk/data_request/cif, or by emailing data_request@ccdc.cam.ac.uk, or by contacting The Cambridge Crystallographic Data Centre, 12 Union Road, Cambridge CB21EZ, UK; fax: + 44 1223 336033.
