# Supplementary material for: PEG-Infiltrated Polyoxometalate Frameworks with Flexible Form-Factors
Source: ACS Mater Lett. 2022 Sep 6;4(10):1937–43. doi: 10.1021/acsmaterialslett.2c00393 (PMC9533303; doi:10.1021/acsmaterialslett.2c00393)
Supplement: Supplementary file 1 — tz2c00393_si_001.pdf [file tz2c00393_si_001.pdf]

# PEG-Infiltrated Polyoxometalate Frameworks with Flexible Form-Factors

Liana S. Alves,[a] Linfeng Chen,[a] Carl E. Lemmon,[a] Milan Gembicky,[a] Mingjie Xu[b] and Alina M. Schimpf[a*]

[a]*Department of Chemistry and Biochemistry, University of California, San Diego, La Jolla, CA 92093, USA*
[b]*Irvine Materials Research Institute, University of California, Irvine, CA 92697, USA*
**Electronic address:* aschimpf@ucsd.edu

**Supplementary Figures and Tables** ........ **S2**
**Table S1.** Crystallographic parameters of PEG-infiltrated $\{P_5W_{30}\}$ frameworks ........ S2
**Figure S1.** Crystal structure of Co-PEG-*Immm* ........ S3
**Figure S2.** Powder X-ray diffraction of Co-PEG-*Immm* in and out of mother liquor ........ S4
**Table S2.** Normalized unit cell volumes for PEG-infiltrated $\{P_5W_{30}\}$ structures ........ S4
**Figure S3.** Powder X-ray diffraction patterns of PEG-infiltrated $\{P_5W_{30}\}$ frameworks ........ S5
**Figure S4.** IR absorption spectra of PEG-infiltrated $\{P_5W_{30}\}$ frameworks ........ S5
**Table S3.** Crystallographic parameters of PEG-infiltrated $\{P_5W_{30}\}$ assemblies ........ S6
**Figure S5.** Crystal structure of $Cu^{2+}$-decorated $\{P_5W_{30}\}$ ........ S7
**Figure S6.** STEM images of rigid film cast from Co-PEG-*Immm* ........ S8
**Figure S7.** SEM images of rigid film cast from Co-PEG-*Immm* ........ S8
**Figure S8.** Powder X-ray diffraction patterns of rigid films with various metals ........ S9
**Figure S9.** Photographs of films attempted from control solution ........ S9
**Figure S10.** Powder X-ray diffraction patterns of PEG-infiltrated $\{P_5W_{30}\}$ ........ S9
**Table S4.** Intramolecular Raman modes of water ........ S10
**Table S5.** Ratio of Raman intensities in PEG-infiltrated $\{P_5W_{30}\}$ samples ........ S10

**Experimental Methods** ........ **S11**
**Table S6.** Chemicals ........ S11
**Synthesis of PEG–Preyssler Composites** ........ S11
**Figure S11.** Powder X-ray diffraction patterns of slow vs rapid syntheses ........ S12
**Formula Determination** ........ S14
**Formation of Films and Gels** ........ S14
**Figure S12.** Flowchart of film and gel formation ........ S14
**Characterization** ........ S15
**Figure S13.** Representative TGA data ........ S15

**References** ........ **S17**

## Supplementary Tables and Figures

**Table S1.** Crystallographic parameters of PEG-infiltrated, transition-metal-bridged $\{P_5W_{30}\}$ frameworks.

| **Bridge** | **Co** | **Mn** | **Fe** | **Ni** | **Zn** |
|---|---|---|---|---|---|
| Empirical formula | $K_2Co_4NaO_{126}P_5W_{30}$ | $K_{1.5}Mn_4NaO_{126}P_5W_{30}$ | $K_2Fe_4NaO_{126}P_5W_{30}$ | $KNi_4NaO_{126}P_5W_{30}$ | $K_2Zn_4NaO_{126}P_5W_{30}$ |
| Formula weight | 8023.26 | 7987.75 | 8010.94 | 7983.28 | 8049.02 |
| Crystal size ($mm^3$) | 0.06 × 0.06 × 0.035 | 0.15 × 0.15 × 0.08 | 0.186 × 0.155 × 0.072 | 0.20 × 0.18 × 0.12 | 0.08 × 0.07 × 0.04 |
| $a$ (Å) | 17.9869(7) | 18.2414(9) | 18.0688(12) | 17.6668 (12) | 17.9607(7) |
| $b$ (Å) | 21.8213(8) | 21.9040(9) | 21.8534(15) | 22.7147(15) | 21.7514(8) |
| $c$ (Å) | 24.8909(10) | 24.9005(10) | 24.8923(17) | 24.8808(17) | 24.8645(10) |
| $\alpha = \beta = \gamma$ (°) | 90 | 90 | 90 | 90 | 90 |
| Volume ($Å^3$) | 9769.6(7) | 9949.2(8) | 9829.1(12) | 9984.6(12) | 9713.8(7) |
| $Z$ | 2 | 2 | 2 | 2 | 2 |
| $\rho_{calc}$ ($g/cm^3$) | 2.727 | 2.666 | 2.707 | 2.655 | 2.752 |
| $\mu$ ($mm^{-1}$) | 18.064 | 17.648 | 17.913 | 17.699 | 18.320 |
| Goodness-of-fit on $F^2$ | 1.029 | 1.032 | 1.098 | 2.700 | 1.033 |
| Reflections collected | 28431 | 29818 | 43927 | 33879 | 38412 |
| Independent reflections | 5040 | 5129 | 5484 | 4979 | 5041 |
| $R_1$ | 0.0598 | 0.0521 | 0.0451 | 0.1664 | 0.0434 |
| $wR_2$ | 0.1963 | 0.1570 | 0.1182 | 0.5925 | 0.1128 |
| Average $M–O_{cluster}$ distance (Å) | 2.06 | 2.15 | 2.10 | 2.18 | 2.06 |
| CSD # | 2145155 | 2145156 | 2145157 | Not deposited | 2145158 |

Radiation: $MoK_\alpha$ ($\lambda$ = 0.71073)
Temperature: 100 K
Crystal system: Orthorhombic
Space group: *Immm*

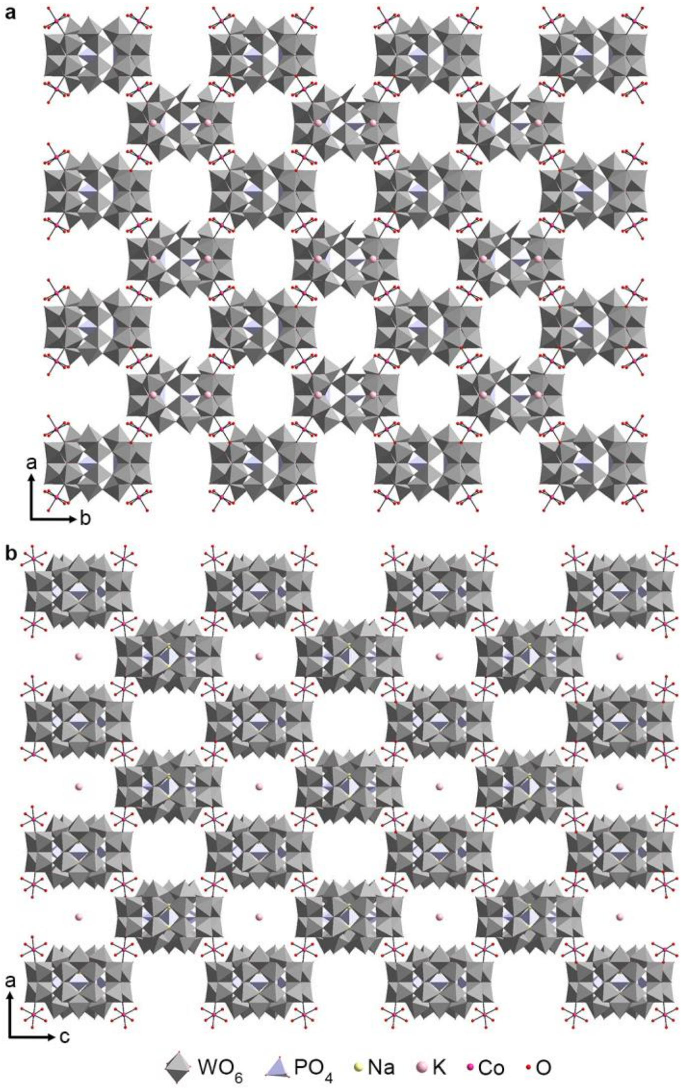


**Figure S1.** Additional views of the crystal structure of Co-PEG-*Immm* showing connectivity in the **(a)** *ab* and **(b)** *bc* planes.

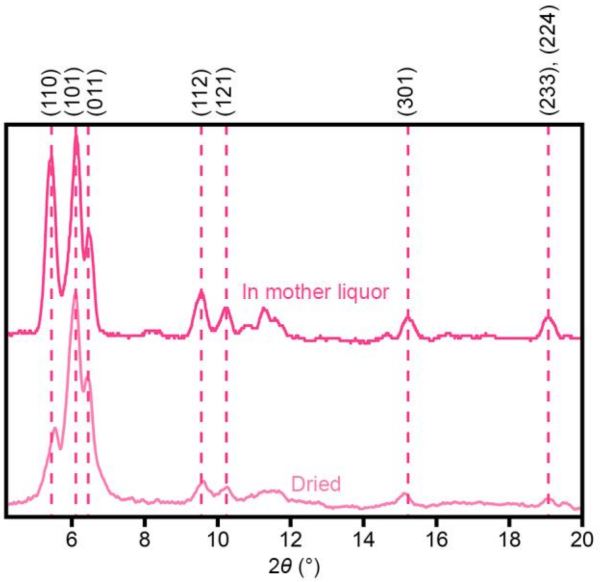


**Figure S2.** Powder X-ray diffraction pattern of crushed crystals of Co-PEG-*Immm* before (top) and after (bottom) removal from the mother liquor. The main reflections do not change position, indicating structural stability.

**Table S2.** Normalized unit cell volumes for PEG-infiltrated $\{P_5W_{30}\}$ structures

| Crystal | $\{P_5W_{30}\}$ per Unit Cell (*Z*) | Unit Cell Volume (Å$^3$) | Calculated Unit Cell Void Volume (*V*, Å$^3$) | Normalized Unit Cell Void Volume (*V/Z*, Å$^3$) | Calculated Void Volume (%) | Maximum # PEG per $\{P_5W_{30}\}^b$ |
|---|---|---|---|---|---|---|
| Co-PEG-*Immm* | 2 | 9770 | 4948 | 2474 | 51 | 4.2 |
| Preyssler-PEG-*C*2/*m* | 4 | 20619 | 13336 | 3334 | 60 | 5.6 |

[a]Calculated using the PLATON routine SQUEEZE[1]

[b]Calculated based on a PEG-400 density of 1.125 g/ml. This would correspond to a molecular radius of 5.2 Å, which is within the range of expected hydrodynamic radii of PEG-400.[2]

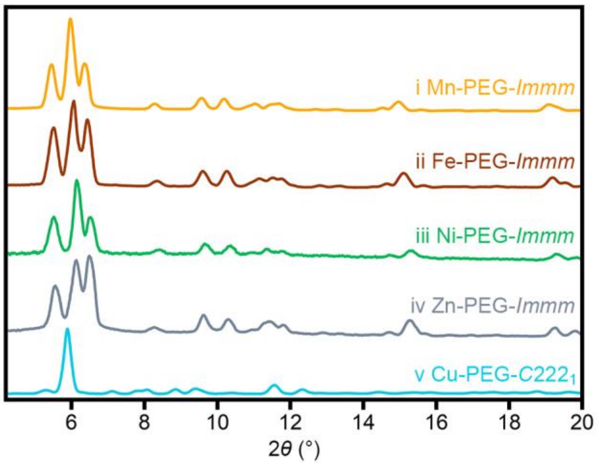


**Figure S3.** Powder X-ray diffraction patterns of PEG-infiltrated $\{P_5W_{30}\}$ crystals synthesized with (i) Mn, (ii) Fe, (iii) Ni and (iv) Zn, which all resulted in isostructural *Immm* frameworks (Table S1). Crystals synthesized with (vi) Cu resulted in crystals of unbridged, decorated clusters (Figure S5).

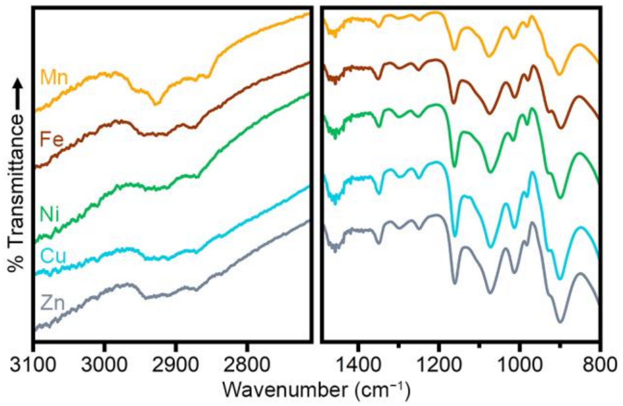


**Figure S4.** IR absorption spectra of PEG-infiltrated $\{P_5W_{30}\}$ crystals synthesized with Mn, Fe, Ni, Cu and Zn. The spectra show inclusion of PEG in all crystals.

**Table S3.** Crystallographic parameters of unbridged, PEG-infiltrated $\{P_5W_{30}\}$ assemblies.

| **Structure** | **Cu-PEG-*C*222₁** | **PEG-{P₅W₃₀}** |
|---|---|---|
| Empirical formula | $Cu_4K_2Na_2O_{125}P_5W_{30}$ | $K_3Na_2O_{110}P_5W_{30}$ |
| Crystal system | Orthorhombic | Monoclinic |
| Space group | $C222_1$ | $C2/m$ |
| Formula weight | 8048.69 | 7593.63 |
| Crystal size (mm$^3$) | 0.219 × 0.169 × 0.092 | 0.124 × 0.09 × 0.068 |
| $a$ (Å) | 25.51900 | 37.868 (3) |
| $b$ (Å) | 34.67400 | 16.8271 (15) |
| $c$ (Å) | 23.30200 | 35.140 (3) |
| $\alpha$ (°) | 90 | 90 |
| $\beta$ (°) | 90 | 94.376(2) |
| $\gamma$ (°) | 90 | 90 |
| Volume (Å$^3$) | 20619 | 22326 (3) |
| $Z$ | 4 | 4 |
| $\rho_{calc}$ (g/cm$^3$) | 2.593 | 2.259 |
| $\mu$ (mm$^{-1}$) | 17.211 | 15.533 |
| Goodness-of-fit on $F^2$ | 0.919 | 1.020 |
| Reflections collected | 10118 | 87965 |
| Independent reflections | 7648 | 16664 |
| $R_1$ | 0.0348 | 0.0531 |
| $wR_2$ | 0.0727 | 0.1614 |
| Average M–O$_{cluster}$ distance (Å) | 2.07 | NA |
| CSD # | 2145159 | 2145160 |

Radiation: MoK$_\alpha$ ($\lambda$ = 0.71073)
Temperature: 100 K

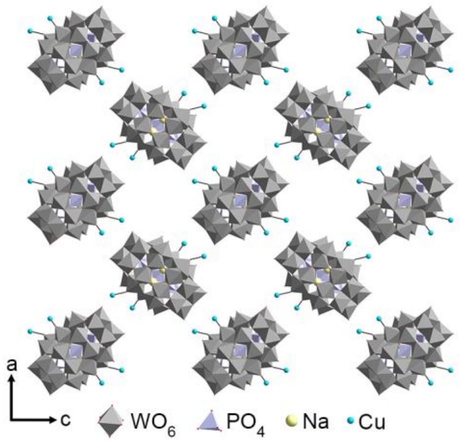


**Figure S5.** Crystal structure of $Cu^{2+}$-decorated {$P_5W_{30}$}. Water and counterions omitted for clarity.

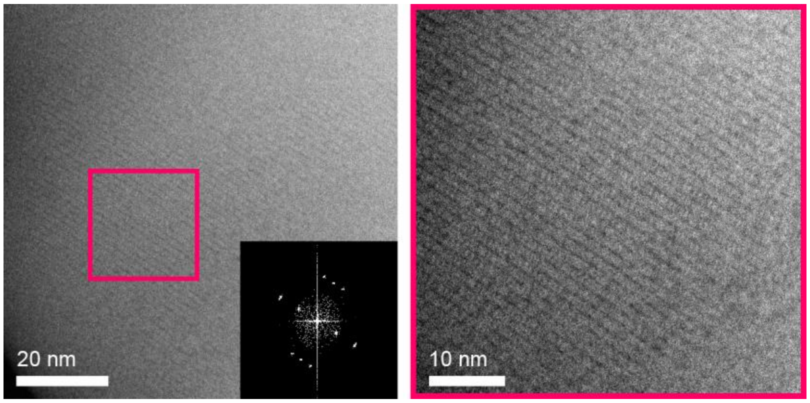


**Figure S6. (a)** MAADF-STEM image and **(b)** Fourier-enhanced image of the area selected from panel a. Inset: Noise-filtered FFT.

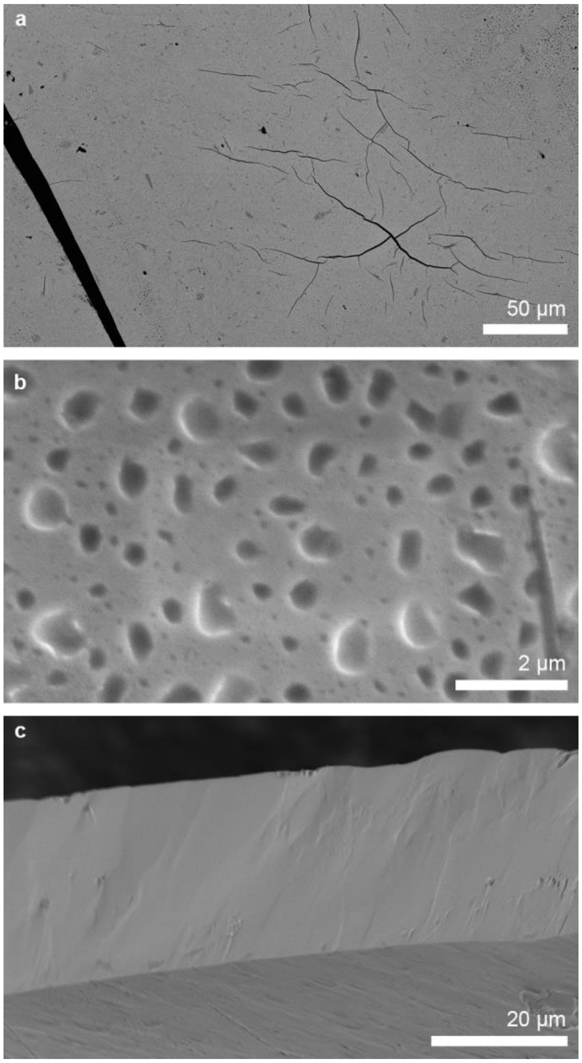


**Figure S7. (a, b)** Top-down and **(c)** cross-sectional SEM images of rigid films cast from Co-PEG-*Immm*.

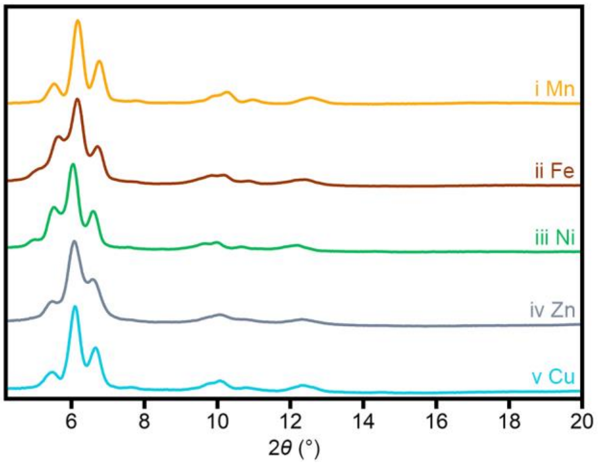


**Figure S8.** Powder X-ray diffraction patterns of rigid films cast from (i) Mn-PEG-*Immm*, (ii) Fe-PEG-*Immm*, (iii) Ni-PEG-*Immm* (iv) Zn-PEG-*Immm* and (v) Cu-PEG-$C222_1$. Although the Cu-films start from a decorated cluster, they adopt the same structure as the other metals once cast into a film.

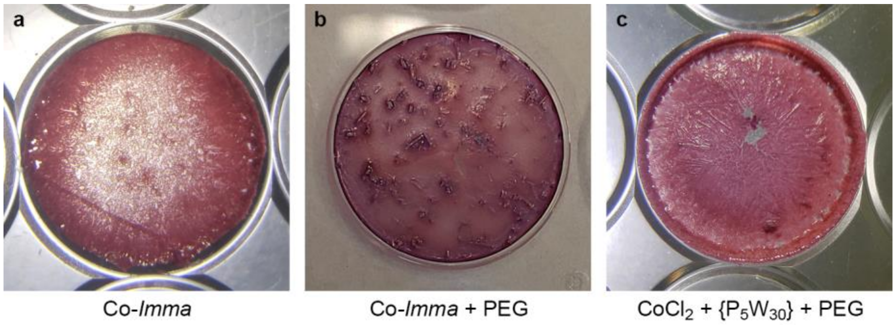


**Figure S9.** Control attempts at casting films from **(a)** Co-*Imma* dissolved in water **(b)** Co-*Imma* dissolved in water and mixed with PEG and **(c)** an aqueous solution of $CoCl_2$ {$P_5W_{30}$} and PEG.

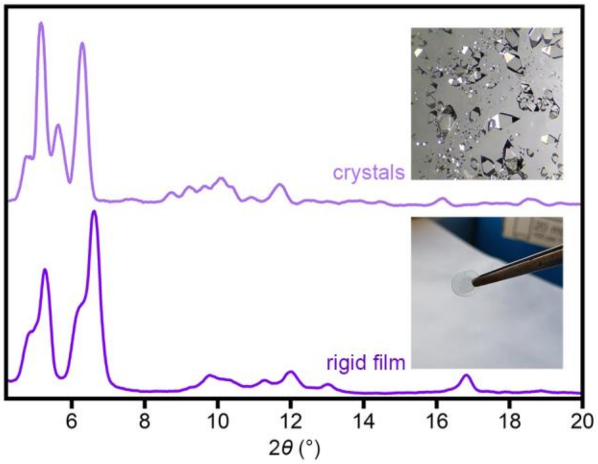


**Figure S10.** Powder X-ray diffraction patterns of (i) PEG-{$P_5W_{30}$} crystals and (ii) a film cast from dissolved PEG-{$P_5W_{30}$}. Insets show photographs of each sample.

**Table S4.** Intramolecular Raman modes of water.

| Category | Mode | Location ($cm^{-1}$) | Reference |
|---|---|---|---|
| Weakly/non-H-bonded | Symmetric O–H stretch | 3612<br>3622<br>3635<br>~3600 | 3<br>4<br>5<br>6 |
| | Asymmetric O–H stretch | 3526<br>3535<br>~3500 | 5<br>4<br>6 |
| Strongly H-bonded | Out-of-phase collective O–H stretch | 3409<br>3411<br>3435<br>~3400 | 5<br>3<br>4<br>6 |
| | In-phase collective O–H stretch | 3236<br>3240<br>3247<br>~3200 | 5<br>3<br>4<br>6 |
| | Fermi resonance | 3045<br>3050<br>~3050 | 3<br>5<br>6 |
| | Isosbestic point | 3460<br>3425 | 4<br>7 |

**Table S5.** Ratios of intensities of the strongly hydrogen-bonded (HB) and weakly/non-hydrogen bonded regions of the water peaks in the Raman spectra of various samples and form-factors.

| Sample | $I_{HB}/I_{NB}$ |
|---|---|
| Gel | 1.6 |
| Flexible film | 1.8 |
| Rigid film | 2.0 |
| Co-PEG-*Immm* crystals | 1.4 |
| PEG-{$P_5W_{30}$} film | 1.2 |
| PEG-{$P_5W_{30}$} crystals | 1.3 |
| Co-*Imma* control | 1.0 |
| Co-*Imma* crystals | 1.0 |

## Experimental Methods

*Chemicals.* Chemicals were purchased from the manufacturers provided in Table S6 and used without further purification.

**Table S6.** Chemicals

| Chemical | Purity | Manufacturer |
|---|---|---|
| Cobalt(II) chloride hexahydrate ($CoCl_2$•$6H_2O$) | 98 % | TCI America |
| Copper(II) chloride dihydrate ($CuCl_2$•$2H_2O$) | >99% | Fischer Chemicals |
| Hydrochloric acid (HCl) | 33.5 % (assay) | EMD Chemicals Inc. |
| Iron(II) chloride tetrahydrate ($FeCl_2$•$4H_2O$) | 98 % | Alfa Aesar |
| Lithium chloride (LiCl) | > 99 % | Fisher Chemical |
| Lithium perchlorate trihydrate ($LiClO_4$•$3H_2O$) | > 95 % | Alfa Aesar |
| Methanol (MeOH) | 99.8 % | Fisher Chemical |
| Manganese(II) chloride tetrahydrate ($MnCl_2$•$4H_2O$) | >95 % | J. T Baker |
| Nickel(II) chloride hexahydrate ($NiCl_2$•$6H_2O$) | 98 % | Spectrum Chemical |
| Phosphoric acid ($H_3PO_4$, 85 %) | 85 % | Fisher Chemical |
| Polyethylene glycol (PEG, MW = 380–420 g/mol) | N/A | TCI America |
| Potassium chloride (KCl) | 99 % | Alfa Aesar |
| Sodium chloride (NaCl) | >99 % | Fisher Chemical |
| Sodium tungstate dihydrate ($Na_2WO_4$•$2H_2O$) | 95% | Spectrum Chemical |
| Zinc(II) chloride ($ZnCl_2$), hydrate | >99% | Alfa Aesar |

**Synthesis of PEG–Preyssler Composites**

*Synthesis of the Preyssler cluster $K_{14-x}Na_x[NaP_5W_{30}O_{110}]$•$15H_2O$ ({$P_5W_{30}$}).* {$P_5W_{30}$} was synthesized following previously reported methods.[8-11] $Na_2WO_4{\cdot}2H_2O$ (9.90 g, 27 mmol), 85% $H_3PO_4$, (7.0 ml), NaCl (1.17g, 20 mmol) and $H_2O$ (21 ml) were added to a 43-ml Teflon-lined acid-digestion vessel. The mixture was stirred until all solids were dissolved at room-temperature, after which the closed vessel was placed in an oven at 125 °C for 20 h. After the reaction was cooled to room-temperature KCl (3 g, 40 mmol) was added and the pale-yellow precipitate was collected by filtration. The product was recrystallized 3 times with 10 ml hot DI water. The final white crystals were collected by filtration and dried for 3 h using vacuum filtration and the purity was verified by collecting a $^{31}$P-NMR spectrum in $D_2O$.

*Synthesis of PEG-infiltrated, Co-bridged frameworks, $H_zLi_yNa_xK_{6-x-y-z}Co_4[NaP_5W_{30}O_{110}]$•mPEG•n$H_2O$ (Co-PEG-Immm).* To a 25 ml round-bottom flask was added 7.0 ml 1 M LiCl (7.0 ml, 1 M, pH 1.0, adjusted by 6 M aqueous HCl), $CoCl_2$•6H2O (156.0 mg, 0.656 mmol) and $K_{12.5}Na_{1.5}[NaP_5W_{30}O_{110}]$•$15H_2O$ (130.0 mg, 0.015 mmol). The flask was equipped with a reflux condenser and the resulting pink solution was heated with rapid stirring to 90 °C for 12 h. After transferring the solution to a 20 ml vial, the solution was concentrated on a hot plate at 80 °C to a volume of ~1.5 ml (~3 h). After the solution was cooled to room temperature polyethylene glycol (PEG, MW ~ 400, 780.0 mg, 1.95 mmol) was added. If precipitates formed upon PEG addition, the solution is not fully cooled. Adjust the solution by one

drop of 1 M LiCl or until the mother liquor is pink. The vial was placed into a sealed 60-ml jar containing 20 ml MeOH. Pink crystals were observed after 3 days and were collected after 2 weeks. Crystals were washed 6 times with 10 ml MeOH using vacuum filtration. Yield: 60 mg, 0.0064 mmol, 42.36% based on {$P_5W_{30}$}. Calculated (experimental) % mass from ICP–MS: 0.11 (0.11) % Li, 0.49 (0.48) % Na, 0.83 (0.79) % K, 2.50 (2.46) % Co, 1.64 (1.58) % P, 58.40 (58.89) % W. Calculated (experimental) % mass $H_2O$ + PEG from TGA mass loss (30–600 °C): 17.38 (16.90) %. Calculated (experimental) % mass from C/H/N combustion: 12.71 (12.24) % PEG, corresponding to 3 PEG per {$P_5W_{30}$}. Formula based on all elemental analyses: $H_{1.5}Li_{1.5}NaK_2Co_4[NaP_5W_{30}O_{110}]$•3PEG •24.5$H_2O$, MW = 9443 g/mol.

Following complete characterization of the single-crystal samples, a "rapid" method was used to synthesize larger sample batches of Co-PEG-*Immm* in less time. To a 500 ml round-bottom flask was added 2.5 ml 12 M LiCl (2.5 ml, 1 M, pH 1.0, adjusted by 6 M aqueous HCl), $CoCl_2$•6H2O (154.5 mg, 0.650 mmol), and $K_{12.5}Na_{0.5}[NaP_5W_{30}O_{110}]$•15$H_2O$ (610.0 mg, 0.074 mmol). Once the salts were fully dissolved, polyethylene glycol (PEG, MW ~ 400, 4.25 g, 10.63 mmol) was added to the flask. The flask was placed in an oil bath at 90 °C, at which point 125 ml MeOH was added with rapid stirring, resulting in immediate formation of a light pink powder. The flask was equipped with a reflux condenser and heated with rapid stirring at 90 °C for 3 h. Pink polycrystalline powders were washed with 2 ml MeOH using vacuum filtration. Powder X-ray diffraction (Figure S11) shows that the rapidly synthesized product adopts the same crystal structure as the single-crystals. Yield: 540.0 mg, 0.061 mmol, 83 % based on {$P_5W_{30}$}. Calculated (experimental) % mass from ICP–MS: 0.08 (0.09) % Li, 0.39 (0.38) % Na, 0.89 (0.90) % K, 3.01 (2.99) % Co, 1.75 (1.45) % P, 62.51 (62.78) % W. Calculated (experimental) % mass $H_2O$ + PEG from TGA mass loss (30–600 °C): 11.41 (11.40) %. Calculated (experimental) % mass from C/H/N combustion: 9.06 (9.03) % PEG, corresponding to 2 PEG per {$P_5W_{30}$}. Formula based on all elemental analyses: $H_{1.5}Li_1Na_{0.5}K_2Co_{4.5}[NaP_5W_{30}O_{110}]$•2PEG •11.5$H_2O$, MW = 8824 g/mol.

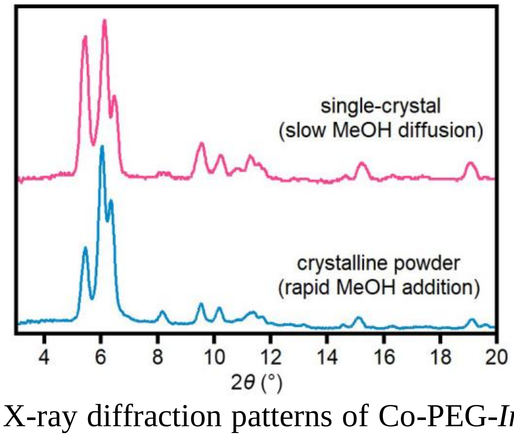


**Figure S11.** Powder X-ray diffraction patterns of Co-PEG-*Immm* synthesized via slow MeOH diffusion and rapid MeOH addition.

*Synthesis of PEG-infiltrated, Mn-bridged frameworks, $H_zLi_yNa_xK_{5-x-y-z}Mn_{4.5}[NaP_5W_{30}O_{110}]$•mPEG•n$H_2O$ (Mn-PEG-Immm).* Mn-bridged frameworks were synthesized following the procedure for Co-bridged frameworks using $MnCl_2$•4$H_2O$ (130 mg, 0.657 mmol), which yielded a pale yellow solution. Light yellow crystals were observed after 2 weeks and collected after 2 months. Yield (one vial): 55 mg, 0.0064 mmol, 42.35% based on {$P_5W_{30}$}. Calculated (experimental) % mass from ICP–MS: 0.24 (0.22) % Li, 0.40 (0.41) % Na,

0.68 (0.60) % K , 2.86 (2.85) % Mn, 1.79 (1.63) % P, 63.70 (63.89) % W. Experimental % $H_2O$ + PEG from TGA mass loss (30–600 °C): 10.01 %. Estimated formula: $Li_3Na_{0.5}K_{1.5}Mn_{4.5}[NaP_5W_{30}O_{110}]$• *m*PEG•*n*$H_2O$, MW = 8658 g/mol.

*Synthesis of PEG-infiltrated, Fe-bridged frameworks, $H_zLi_yNa_xK_{5-x-y-z}Fe_{4.5}[NaP_5W_{30}O_{110}]$•mPEG•n$H_2O$ (Fe-PEG-Immm).* Fe-bridged frameworks were synthesized following the procedure for Co-bridged frameworks using $FeCl_2$•4$H_2O$ (130 mg, 0.604 mmol), which yielded a pale yellow solution. Dark brown crystals were observed after 3 days and collected 1.5 weeks. Yield (one vial): 64 mg, 0.0061 mmol, 40.56% based on {$P_5W_{30}$}. Calculated (experimental) % mass from ICP–MS analysis: 0.13 (0.15) % Li, 0.33 (0.33) % Na, 0.74 (0.74) % K , 2.39 (2.28) % Fe, 1.47 (1.49) % P, 52.43 (52.48) % W. Experimental % $H_2O$ + PEG from TGA mass loss (30–600 °C): 25.78 %. Estimated formula: $H_{0.5}Li_2K_2Na_{0.5}Fe_{4.5}[NaP_5W_{30}O_{110}]$•*m*PEG•*n*$H_2O$, MW = 10520 g/mol.

*Synthesis of PEG-infiltrated, Ni-bridged frameworks, $H_zLi_yNa_xK_{5-x-y-z}Ni_{4.5}[NaP_5W_{30}O_{110}]$•mPEG•n$H_2O$ (Ni-PEG-Immm).* Ni-bridged frameworks were synthesized following the procedure for Co-bridged frameworks using $NiCl_2$•6$H_2O$ (156 mg, 0.656 mmol), which yielded a green solution. Pale green crystals were observed after two weeks and collected after three weeks. Yield (one vial): 60 mg, 0.0063 mmol, 42.13% based on {$P_5W_{30}$}. Calculated (experimental) % mass from ICP–MS analysis: 0.26 (0.25) % Li, 0.36 (0.35) % Na, 0.41 (0.47) % K, 2.78 (2.81) % Ni, 1.63 (1.47) % P, 58.08 (58.15) % W. Experimental % $H_2O$ + PEG from TGA mass loss (30–600 °C): 17.94 %. Estimated formula $Li_{3.5}Na_{0.5}KNi_{4.5}[NaP_5W_{30}O_{110}]$•*m*PEG•*n*$H_2O$, MW = 9495 g/mol.

*Synthesis of PEG-infiltrated, Zn-bridged frameworks, $H_zLi_yNa_xK_{6-x-y-z}Zn_4[NaP_5W_{30}O_{110}]$•mPEG•n$H_2O$ (Zn-PEG-Immm)*. Zn-bridged frameworks were synthesized following the procedure for Co-bridged frameworks using $ZnCl_2$ (100 mg, 0.734 mmol), which yielded a colorless solution. Additionally, aqueous LiCl was replaced with aqueous $LiClO_4$ (2.0 ml, 0.05 M, pH 1.0, adjusted by 6 M aqueous HCl). Colorless crystals were observed after two weeks and collected after three weeks. Yield (one vial): 30 mg, 0.0033 mmol, 21.90% based on {$P_5W_{30}$}. Calculated (experimental) % mass from ICP–MS analysis: 0.00 (0.14) % Li, 0.76 (0.73) % Na, 1.50 (1.50) % K, 2.86 (2.97) % Zn, 1.70 (1.62) % P, 60.39 (60.37) % W. Experimental % $H_2O$ + PEG from TGA mass loss (30–600°C): 13.53 %. Estimated formula: $H_{0.5}Na_2K_{3.5}Zn_4[NaP_5W_{30}O_{110}]$•*m*PEG•*n*$H_2O$, MW = 9133 g/mol.

*Synthesis of PEG-infiltrated, Cu-decorated Preyssler, $H_zLi_yNa_xK_{6-x-y-z}Cu_4[NaP_5W_{30}O_{110}]$•mPEG•n$H_2O$ (Cu-PEG-$C222_1$).* Cu-bridged frameworks were synthesized following the procedure for Co-bridged frameworks, using $CuCl_2$•6$H_2O$ (104 mg, 0.610 mmol), which yielded a brown solution. Additionally, aqueous LiCl was replaced with aqueous $LiClO_4$ (2.0 ml, 0.5 M, pH 1.0, adjusted by 6 M aqueous HCl). Pale blue, colorless, and brown crystals were observed after 1 week and collected after three weeks. Yield (one vial): 60 mg, 0.0060 mmol, 39.68% based on {$P_5W_{30}$}. Calculated (experimental) % mass from ICP–MS analysis: 0.00 (0.00) % Li, 0.34 (0.39) % Na, 0.78 (0.86) % K, 2.52 (2.58) % Cu, 1.54 (1.43) % P, 54.72 (54.64) % W. Experimental % $H_2O$ + PEG from TGA mass loss (30–600 °C): 22.65 %. Estimated formula: $H_{3.5}Na_{0.5}K_2Cu_4[NaP_5W_{30}O_{110}]$•*m*PEG•*n*$H_2O$ , MW = 10080 g/mol.

*Synthesis of PEG-infiltrated Preyssler, $H_zLi_yNa_xK_{14-x-y-z}[NaP_5W_{30}O_{110}]$•*mPEG•*n*$H_2O$ *(PEG-{$P_5W_{30}$})*. Composites were synthesized following the procedure for Co-bridged frameworks in absence of transition metal which yielded a colorless solution. Colorless crystals were observed after one day and collected after 1 week. Yield (one vial): 130 mg, 0.013 mmol, 86.67 % based on {$P_5W_{30}$}. Calculated (experimental) % mass from ICP–MS analysis: 0.42 (0.42) % Li, 0.92 (0.96)

% Na, 1.37 (1.35) % K, 1.55 (1.53) % P, 55.16 (54.78) % W. Calculated (experimental) % $H_2O$/PEG from TGA mass loss (30–600 °C): 22.98 (23.48) %. Calculated (experimental) % mass from C/H/N combustion: 20.00 (20.48) % PEG corresponding to 5 PEG per $\{P_5W_{30}\}$. Formula based on all elemental analyses: $H_{1.5}Li_6Na_3K_{3.5}[NaP_5W_{30}O_{110}]$•5PEG•16.5$H_2O$, MW = 9999 g/mol.

**Formula Determination**

For all the materials presented above, Li, K, M (M = Mn, Fe, Co, Ni, Cu or Zn), Na, P and W contents were determined using inductively coupled plasma mass spectrometry (ICP–MS) and the total mass % of $H_2O$ + PEG was determined using the % mass lost during thermogravimetric analysis (TGA, 30–600 °C). For Co-PEG-*Immm* and Preyssler-PEG-*C*2/*m*, the % PEG was determined using C/H/N combustion and was corroborated using quantitative NMR spectroscopy. Water was assumed to be the remainder of the mass lost during TGA. Water was assumed to be the remainder of the mass lost during TGA. Further details are provided in the Characterization Section.

**Formation of Films and Gels**

Film and gel formation is outlined in Figure S12

*Films.* Co-PEG-*Immm* (60 mg) was dissolved in 1000 µl $H_2O$ and 200 µl of this solution was dispensed on a 96-well culture plate lid. Water was allowed to evaporate and free-standing films formed after ~12 h. The mechanical properties of films formed this way are dependent on the humidity during evaporation. Films formed when the relative humidity (RH) is below ~60% are rigid/brittle and those formed above RH ~60% are flexible. The films could be switched between flexible and rigid states by placing in a controlled humidity environment or by driving off water using heat. This allowed switching within ~1 min.

*Gels.* Co-PEG-*Immm* (1 g) was dissolved in 100 µl $H_2O$ and the solution was heated at 50 °C for ~3 min to promote full dissolution.

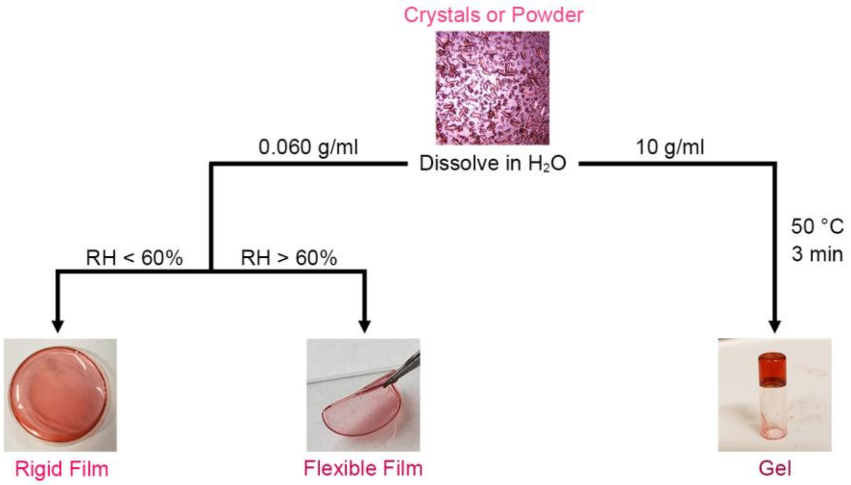


**Figure S12.** Flowchart of film and gel formation.

## Characterization

*Basic characterization and formula determination.* Infrared absorption spectra were collected using an Agilent Cary 630 ATR-FTIR. NMR spectra used to confirm cluster purity were collected using a Bruker AVA spectrometer (300 MHz).

ICP–MS was collected using a Thermo iCAP RQ ICP–MS. The samples were digested in a 1:1 mixture of 85% $HNO_3$ (Optima grade, Sigma Aldrich) and 30 % $H_2O_2$ (Trace metal grade, Sigma Aldrich) with sonication for 90 min. TGA was collected using a Perkin-Elmer STA 6000 under $N_2$ with a heating-rate of 5 °C/s. Representative TGA data are provided in Figure S13. C/H/N analysis was conducted by NuMega Resonance Labs using a Perkin Elmer PE2400-Series II, CHNS/O.

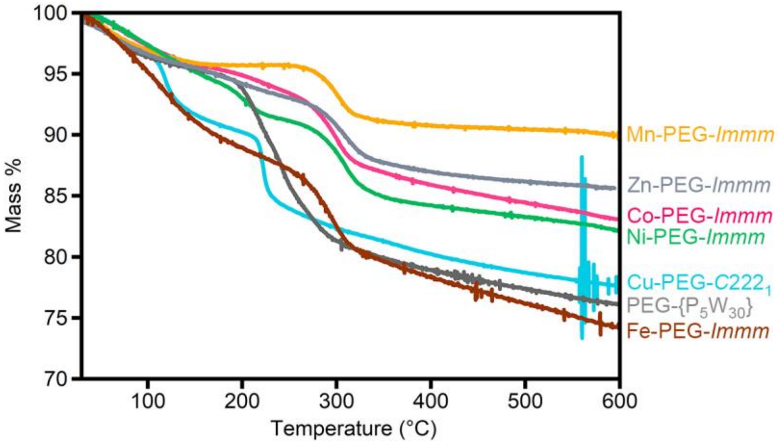


**Figure S13.** Representative TGA data.

For quantitative NMR measurements, Co-PEG-*Immm* crystals (20 mg) were dissolved in ~700 μl $D_2O$ with an internal standard of DMSO (20 mg). Spectra were collected at room-temperature on a Varian VX 500 Spectrometer with an inverse gated decoupling pulse sequence to remove NOE signal (delay time = 360 s). The resonance of the repeating $CH_2$ of PEG ($\delta$ = 69.41 ppm) was integrated from 69.783 to 68.883 ppm. These experiments yielded 11.67 % mass PEG (compared to 12.24% by C/H/N combustion).

*Single-crystal X-ray diffraction.* Single-crystal X-ray diffraction was performed using a Bruker APEX-II Ultra CCD diffractometer equipped with Mo Kα radiation ($\lambda$= 0.71073 Å). Crystals were mounted on a Cryoloop with Fomblin Y oil. Data were collected in a nitrogen gas stream at 100(2) K using Φ and ω scans. Crystal-to-detector distance was 45 mm and exposure time was 10 s per frame using a scan width of 0.75°. Indexing and unit cell refinement indicated a primitive, orthorhombic lattice. The data were integrated using the Bruker SAINT software program and scaled using the SADABS software program. Solution by direct methods (SHELXT) produced a complete phasing model consistent with the proposed structure. All nonhydrogen atoms were refined anisotropically by full-matrix least-squares (SHELXL-2014).[12] For highly disordered water molecules, the PLATON routine SQUEEZE was used to account for the corresponding electrons as a diffuse contribution to the overall scattering without specific atom positions.[1] In order to model disorder structures ISOR, EADP and RIGU commands have been used. For Mn-PEG-*Immm*, Fe-PEG-*Immm*, Co-PEG-*Immm*, Ni-PEG-*Immm* and Zn-PEG-*Immm*, the position of all countercations have not been assigned due to high disorder. All I*mmm* deposited structures and PEG-{$P_5W_{30}$} contain two disordered configurations of the inner tungsten belt of

$\{P_5W_{30}\}$. This disorder is removed to show a simplified structure in Figures 1a and S1. PLATON's CALC SOLV[1] was performed on Co-PEG-*Immm* and Preyssler-PEG-C*2*/*m* to find the total accessible solvent in the void volume percent.

The Ni-PEG-*Immm* data has not been deposited to the CCDC/FIZ Karlsruhe database. Although the structure is isomorphous with the other PEG-*Immm* structures, Ni-PEG-*Immm* has high diffuse scattering and thermal motion, in which classical refinement values have a high *R*-value of ~16%. A simple disorder model did not improve the structural solution. Three full data sets were screened and yielded similar diffuse scattering with motion along the *b* direction due to hydrogen-bonding in the structure, the soft serial arrangement of water, and the presence of PEG. The cluster shows two possible disordered confirmations, one of which is rotated and shifted, most likely due to the flexibility of the Ni bridging ion. Furthermore, attempts at lowering the symmetry did not improve the disorder model. The structure was thus not deposited to database, but the CIF is provided as supplemental information.

In Cu-PEG-*C*222$_1$, Cu1 is disordered and modeled with two positions at half occupancy. Cu1A is coordinated by one $\{P_5W_{30}\}$, while Cu1B is not coordinated by $\{P_5W_{30}\}$. Although only 4 water molecules could be assigned near Cu1, the blue color of the framework indicates octahedral coordination of $Cu^{2+}$. It is thus assumed that Cu1A and Cu1B share some disordered water in their environment.

*Powder X-ray diffraction.* For powder X-ray diffraction measurements, crystals were crushed between two glass slides and placed on a mounting loop. Data were collected on a Bruker K3 Kappa Vantec 500 diffractometer equipped with Cu Kα radiation ($\lambda$ = 1.54184). The measurements were performed in transmission mode with detector distance of 200 mm and 3 frames collected $\varphi = 0°$ and 45°. The first, second, and third frames were centered at $2\theta = 10°$, 22° and 34°, respectively. The diffraction rings obtained from the three frames were overlaid together and radially integrated in Diffrac.EVA V.4.2.2 (Bruker).

*Scanning Transmission Electron microscopy (STEM).* A rigid film of Co-PEG-*Immm* was sonicated in ethanol and the suspension was pipetted onto a lacey carbon grid. Medium angle annular dark field (MAADF) images were acquired using JEOL Grand ARM aberration-corrected STEM under 300kV. The enhanced image was generated after noise-filtering the fast Fourier transform of a selected area of the original image.

*Scanning Electron Microscopy (SEM).* A rigid film of Co-PEG-*Immm* was affixed to the SEM sample holder using carbon tape. Images were collected on a FEI Apreo SEM with an operating voltage of 5.00 kV and emission current of 0.80-0.10 nA.

*Rheology*. Rheological measurements conducted using a Discovery HR-3 rheometer (TA instruments). Samples were prepared fresh and measured within 1 hr. The gel was placed on the surface of a 40-mm sandblasted plate and with a loading gap of 45000 µm. The temperature was set at 20 °C using a Peltier heating system. For oscillatory tests, an amplitude sweep was carried out at strains of 1.0–100 % with an angular frequency of 10 rad/s. The frequency sweep was made from the linear region of the amplitude state from the oscillatory test to determine the linear viscoelastic range. The frequency sweep was performed at 2.6 % strain at 20.0–300 rad/s.

*Raman spectroscopy*. Raman spectra were collected using a Thermo Scientific™ DXR™3 SmartRaman with an extended gradient of 6000 to 50 $cm^{-1}$. Using a YAG laser that had single-mode operation at 532 nm with an output power of 10 mW, the spectra were measured over the frequency range between 50 and 4000 $cm^{-1}$. The laser beam was focused using an optical system and open beam expanded onto the surface of the sample at a spot with a 50 µm diameter.

**References**


1. Spek, A. L. PLATON SQUEEZE: A Tool for the Calculation of the Disordered Solvent Contribution to the Calculated Structure Factors. *Acta Crystallogr. C* **2015,** *71*, 9.
2. Dohmen, M. P. J.; Pereira, A. M.; Timmer, J. M. K.; Benes, N. E.; Keurentjes, J. T. F. Hydrodynamic Radii of Polyethylene Glycols in Different Solvents Determined From Viscosity Measurements. *J. Chem. Eng. Data* **2008,** *53*, 63.
3. Kudo, K.; Ishida, J.; Syuu, G.; Sekine, Y.; Ikeda-Fukazawa, T. Structural Changes of Water in Poly(vinyl alcohol) Hydrogel During Dehydration. *J. Chem. Phys.* **2014,** *140*, 044909.
4. Walrafen, G. E. Raman Spectral Studies of the Effects of Temperature on Water Structure. *J. Chem. Phys.* **1967,** *47*, 114.
5. Sekine, Y.; Ikeda-Fukazawa, T. Structural Changes of Water in a Hydrogel During Dehydration. *J. Chem. Phys.* **2009,** *130*, 034501.
6. Ratajska-Gadomska, B.; Gadomski, W. Water Structure in Nanopores of Agarose Gel by Raman Spectroscopy. *J. Chem. Phys.* **2004,** *121*, 12583.
7. Whiteman, D. N.; Walrafen, G. E.; Yang, W. H.; Melfi, S. H. Measurement of an Isosbestic Point in the Raman Spectrum of Liquid Water by Use of a Backscattering Geometry. *Appl. Optics* **1999,** *38*, 2614.
8. Alizadeh, M. H.; Harmalker, S. P.; Jeannin, Y.; Martinfrere, J.; Pope, M. T. A Heteropolyanion with Fivefold Molecular Symmetry That Contains a Nonlabile Encapsulated Sodium-Ion - the Structure and Chemistry of $[NaP_5W_{30}O_{110}]^{14-}$. *J. Am. Chem. Soc.* **1985,** *107*, 2662.
9. Hayashi, A.; Haioka, T.; Takahashi, K.; Bassil, B. S.; Kortz, U.; Sano, T.; Sadakane, M. Cation Effect on Formation of Preyssler-Type 30-Tungsto-5-Phosphate: Enhanced Yield of Na-encapsulated Derivative and Direct Synthesis of Ca- and Bi-Encapsulated Derivatives. *Z. Anorg. Allg. Chem.* **2015,** *641*, 2670.
10. Turo, M. J.; Chen, L.; Moore, C. E.; Schimpf, A. M. $Co^{2+}$-Linked $[NaP_5W_{30}O_{110}]^{14-}$: A Redox-Active Metal Oxide Framework with High Electron Density. *J. Am. Chem. Soc.* **2019,** *141*, 4553.
11. Chen, L.; Turo, M. J.; Gembicky, M.; Reinicke, R., A.; Schimpf, A. M. Cation-Controlled Assembly of Polyoxotungstate-Based Coordination Networks. *Angew. Chem. Int. Edit.* **2020,** *59*, 16609.
12. Sheldrick, G. SHELXT – Integrated Space-Group and Crystal-Structure Determination. *Acta Crystallogr. A* **2015,** *71*, 3.
